# The Environmental Effects on Virulence Factors and the Antifungal Susceptibility of *Cryptococcus neoformans*

**DOI:** 10.3390/ijms22126302

**Published:** 2021-06-11

**Authors:** Mahek Momin, Ginny Webb

**Affiliations:** Division of Natural Sciences and Engineering, University of South Carolina Upstate, 800 University Way Spartanburg, Spartanburg, SC 29303, USA; mominm@email.uscupstate.edu

**Keywords:** *Cryptococcus*, antifungal, virulence factor, environmental effects

## Abstract

*Cryptococcus neoformans* is a facultative intracellular pathogen responsible for fungal meningoencephalitis primarily in immunocompromised individuals. It has become evident the pathogenicity of *C. neoformans* is dependent on the fungal cell’s environment. The differential expression of virulence factors, based on the cell’s environmental conditions, is one mechanism allowing for the environmental control of the pathogenic ability of *C. neoformans*. Here, we discuss how these virulence factors (including melanin, the polysaccharide capsule, and Antiphagocytic protein 1) have been shown to be differentially expressed dependent on the cell’s environment. The genetics and signaling pathways leading to the environmental-dependent regulation of virulence factors will also be examined. Susceptibility to antifungal therapeutics is also regulated by the environment, and thus affects the pathogenic abilities of *C. neoformans* and disease outcomes. This review will also examine the role of the *C. neoformans*’s environment on antifungal susceptibilities, and the genetics and signaling pathways responsible for these susceptibility alterations. By examining the complex interplay between the environment and the pathogenicity of *C. neoformans*, we have a better understanding of the intricacies of the pathogen–environment interaction and how to exploit this interaction to develop the most effective treatment protocols.

## 1. Introduction

Fungal meningoencephalitis is a life-threatening disease, which has represented a significant public health burden for several decades, especially in resource-limited countries [1]. The pathogenic encapsulated yeast, *Cryptococcus neoformans*, has been recognized as the most common instigator of the disease and has been impacting many immunocompromised populations, especially individuals with acquired immunodeficiency syndrome (AIDS) [2]. There are almost 220,000 new cases and 181,000 deaths occurring worldwide annually, with most of these cases in sub-Saharan Africa [1,3]. *Cryptococcus neoformans* can be found naturally in soil, bird droppings, and trees [4]. Desiccated yeasts or spores from the environment can be inhaled into the host, and if not cleared can result in Cryptococcosis. In an immunocompromised host, unable to adequately clear the fungus, it can disseminate to the central nervous system, resulting in life-threatening meningitis [5]. This fungus produces several virulence factors that aid in its pathogenicity. These virulence factors include a polysaccharide capsule, Antiphagocytic protein 1 (App1), and melanin [6,7]. Environmental parameters impact virulence factors of the fungus, which enables *C. neoformans* to effectively adjust to inconsistent environments [8]. This is especially important as the fungal cell transitions from its environmental reservoir to the human host. The common therapeutic protocol for Cryptococcosis involves the antifungals fluconazole (FLU), flucytosine (5-FC) and amphotericin B (AMB) [9]. The susceptibility of *C. neoformans* to these treatments has been decreasing, and is variable based on the environment and expression profile of the fungal cell. In this review, we will discuss the regulation of virulence factors and antifungal susceptibilities by environmental conditions. The signaling and mechanisms responsible for these regulatory processes will also be reviewed.

## 2. Environmental Effects on Virulence Factors

*Cryptococcus neoformans* is omnipresent in the environment and frequently found in soil contaminated with bird droppings. An outlet for the cryptococcal cells to return to the environment is through the decay of an infected host or host body part [5]. The yeast is then inhaled into the human host to initiate infection. To resist the host immune response, *C. neoformans* produces numerous virulence factors, such as a polysaccharide capsule, melanin formation, and App1 [6,7]. These virulence factors allow for the fungus to survive environmental changes through a variety of signaling pathways to initiate their growth and resistance. Many of these virulence factors have been shown to be regulated based on the fungal cell’s environment. These findings are important as they imply that the expression of virulence factors may be different in the natural environment compared to the human host. The alteration of virulence factor expression can affect the host–pathogen interaction, the host’s immune response, and antifungal susceptibilities.

### 2.1. Capsule

The polysaccharide capsule that surrounds *C. neoformans* is developed to prevent phagocytosis from occurring. Immune cells do not ingest encapsulated *C. neoformans* without antibody or complement opsonization. The capsule limits the immune response that signals for antibody production and the complement proteins are also depleted, leading to minimal opsonization and therefore decreased phagocytosis [10]. Various environmental conditions have been shown to promote capsule growth, such as CO_2_ concentration, sera, pH levels, temperature, and iron acquisition. Interestingly, capsule size has been shown to be increased in the mammalian lung and decreased in the mammalian brain [11,12,13]. In a study where mammalian serum and CO_2_ were tested to see their impact on capsule growth in *C. neoformans*, it was discovered that there was variability between the strains in their response to either serum or CO_2_. Some strains were found to require both environmental stimuli to induce capsule formation [14].

Additionally, it has been reported that capsule induction can also be pH- and temperature-dependent. Results have indicated that in media where capsule sizes were found to be increased, the pH was greater than 7. After adjusting the pH to 5.6, it was found that the serum did not sufficiently increase the size of the capsule. This result indicates that environments with a pH less than 7 inhibited capsule growth [14]. Additionally, previous studies further support this as capsule growth was indicated in more alkaline pH mediums [15]. Similarly, in another study that evaluated the impact of pH on *C. neoformans* cells, maximum capsule growth occurred in more alkaline environments, hence indicating capsule growth in high pH [16]. Host phagocytes are able to acidify the phagosomal compartment after ingestion of microbes; however, the capsule of *C. neoformans* can interfere with phagosomal acidification, serving as a buffer [17].

Upon evaluation of the role of temperature in the capsule development, there was found to be a larger capsule volume at 37 °C. Interestingly, the cell’s regulation of the size of the capsule and the outcome of the infection was strain-specific [18]. Intriguingly, iron has also been found to be an essential nutrient for pathogen proliferation in mammalian hosts. In Vps23 and Rim101 mutants of *C. neoformans*, there was found to be an iron-related growth defect, which resulted in a reduced cell-associated capsule, increased capsule shedding, and increased sensitivity to alkaline pH [19]. Iron has been found to upregulate *C. neoformans* capsule virulence, as its exacerbation in the host has allowed for the prevalence of cryptococcosis [20].

### 2.2. Melanin

Melanin is a polymer that is produced through catecholamine oxidation, which accrues in the cell wall of *C. neoformans*. Melanin aids in protecting against the oxidants that are produced by immune cells and decreases the permeability of the cell wall. Melanin coats the cell wall of *C. neoformans* to assist in the survival of the fungus. The pathogenic fungus also produces melanin within its cell wall to fight against infection, and can be induced secondary to external stresses such as exposure to stress, temperature, and heavy metals. Additionally, once inside the host, macrophages release bursts of reactive oxygen species (ROS) and reactive nitrogen species (RNS) into the phagosomes to create a highly oxidative environment, which is fatal to most engulfed organisms. However, it has been found that melanin production is reduced when exposed to oxidative stress and increased in the presence of moderate concentrations of RNS in the surrounding environment. Growth at 37 °C also reduced melanin production [21]. Similarly, in a recent study by Zhou et al. [22], it has been found that the presence of ROS/RNS during fungal infection is a major determinant of the outcome of the disease.

Melanin is produced by the pigment laccase, and laccase synthesis is induced by many factors, which includes the metabolism of copper and other heavy metals. In experiments containing L-dopa, which is a laccase substrate, copper sulfate was incorporated in varying concentrations in which cultures of *C. neoformans* showed increased melanization in comparison to cultures without the addition of copper sulfate. The clinical and environmental samples indicated a great difference, which indicated that the presence of copper sulfate at the growth site of the samples leads to an increase in melanization, and thus ultimately led to enhanced virulence of *Cryptococcus neoformans* [23]. Upon exposure to environmental silver nitrate (AgNO3), which is a highly toxic compound for both bacteria and fungi, it was indicated that the melanization of *C. neoformans* reduces the susceptibility to the toxic heavy metal. This finding suggests that the melanization of the fungus has a critical role in protecting it against heavy metal toxicity, thus allowing for the survival of *C. neoformans* [24]. In the presence of exogenous diphenolic compounds, such as L-dopa, *C. neoformans* produces melanin through laccases (Lac1 and Lac2). When laccase is expressed, laccases are loaded into secretory vesicles and placed into the cell wall using chitin as an anchoring molecule, or they are secreted extracellularly. A comprehensive regulatory mechanism of melanin remains unidentified; however, prior studies have indicated the following two major signaling pathways: the cyclic AMP/protein kinase A (cAMP/PKA) and high-osmolarity glycerol response (HOG) pathways, which aid in the biosynthesis of the melanin production of *C. neoformans* [25]. In all, melanin has been demonstrated to be affected by ROS/RNS, temperature, and heavy metals. It has been difficult to determine the expression of melanin during infection, but studies have utilized techniques that conclude melanin may be expressed in the mammalian host [26].

### 2.3. Antiphagocytic Protein 1

Antiphagocytic protein 1 (App1) is a cryptococcal protein that is secreted extracellularly and found in the serum of infected patients. Because *C. neoformans* is inhaled into the lungs to initiate infection, host alveolar macrophages are vital to the immune response against *C. neoformans*. App1 was discovered as a protein downstream of inositol phosphoryl ceramide synthase (Ipc1), which inhibits phagocytosis of the yeast. App1 inhibits both attachment and ingestion of the yeast [27]. It was discovered that App1 inhibits phagocytosis through a complement-mediated mechanism, specifically by binding to complement receptor 3 [28]. Interestingly, it was discovered that the effect of App1 on Cryptococcosis is dependent on the immune state of the host. In an immunocompetent host, the inhibition of phagocytosis results in unchecked fungal growth and dissemination to the CNS. In an immunocompromised host, the inhibition of phagocytosis actually leads to an increase in survival. This is because when the yeast is phagocytosed, the macrophage may not be activated to kill the pathogen, due to an inadequate T cell response. Therefore, phagocytosed *C. neoformans* in an immunocompromised host actually grows inside the macrophage faster than it would extracellularly, and is hidden from the remaining immune responses. This allows for quicker dissemination and progression of the disease. Therefore, inhibition of phagocytosis in an immunocompromised host actually slows down disease progression [27].

App1 has been found to be differentially expressed based on the fungal cell environment [6]. Specifically, App1 is upregulated in conditions of the human host lung, including 37 °C, 5% CO_2_, and low glucose. This is important because the instrumental role of App1 in the host–pathogen interaction occurs in the lung environment. App1 expression appears to be regulated throughout the infectious process, dependent on the need of the protein in each host environment. In tested fluids, App1 expression was the highest in the bronchoalveolar lavage (BAL), where the protein could be most influential, and lowest in blood serum, where the protein would have less of an effect [6]. In addition to these environmental parameters, older age of Cryptococcal cells also increased App1 expression [29]. The mechanism behind App1’s regulation is not fully known. Several potential signaling pathways were tested, based on known signaling in *C. neoformans*, as well as similar pathways in Candida albicans and Saccharomyces cerevisiae. App1’s differential expression appears to be lessened in Δgpa1 and Δsnf1 strains, suggesting these genes and their respective pathways may be involved [6]. App1 is another example of a virulence factor of *C. neoformans* that is differentially expressed based on environmental conditions, leading to direct effects on the host–pathogen interaction and disease progression or containment.

### 2.4. Urease and Phospholipase

Urease is an enzyme responsible for the conversion of urea to ammonia and carbon dioxide. Urease had been discovered to contribute to the virulence of several other pathogens and was also shown to be a virulence factor of *C. neoformans* [30]. Urease affects the outcome of internalized *C. neoformans* cells by affecting the fitness of the yeast in the phagosome and delaying replication. This effect could increase the dissemination of the yeast in the host [31]. Urease expression was shown to be regulated by the nitrogen availability in the cells’ environment [32]. Studies on urease and the regulation of its expression are emerging, but it has been shown that VPH1 plays a role, as there was no urease activity in a VPH1 mutant [33].

Phospholipases are enzymes that hydrolyze phospholipids. PLB1 has been identified as a virulence factor of *C. neoformans* [34]. Phospholipase has been shown to maintain the fungal cell wall. Exposure to heat causes Plb1 to accumulate in the cell wall [35]. Both of these enzymes are newly discovered virulence factors of *C. neoformans*, and more research is needed to fully know how the cell’s environment may affect their expression.

### 2.5. Host Macrophage–Cryptococcus Interactions

The ability of *C. neoformans* to resist what should be the hosts’ first line of defense, phagocytosis by macrophages, is critical to analyze and understand. This interaction plays a significant role in the outcome of the disease [36]. Although the yeast is engulfed by the host phagocytes, it still has the ability to survive and replicate within the phagocytes, instead of being destroyed. This is one of the most critical parts of the *C. neoformans* infectious process, aiding in its virulence. After the analysis of interactions between the host cells and *C. neoformans*, it has been found that human THP-1 cells may be readily differentiated into macrophage-like cells in culture, and, upon the assay, they demonstrated a high phagocytic capability, without activation by antibodies or LPS. The THP-1 cells were found to be sensitive to *C. neoformans*, as they can detect alterations in the capsule within less than two hours of capsule formation by the conditions of the host. The number of cryptococcal cells added to each host well in the assay, and the uptake or adherence by phagocytes, was noted to have a linear relationship. It was discovered that adherence begins to level off close to 30 min after exposure. As early as an hour after capsule formation, reduced adherence by phagocytes was noted. These results indicate that adherence paves the way for internalization by the host phagocytes of the *C. neoformans* cells [37].

It has been demonstrated that host macrophages are essential for the control of the pathogen, as their depletion was found to have a deteriorating effect on any restriction of fungal burden. The destruction of macrophages, even after the controlling of the infection, still led to increased fungal burden, which indicates that they continue to play a significant role in host–pathogen interactions even after the onset of the infection. Interestingly, there was found to be a high level of intracellular proliferation between 2 and 8 h of infection, which could explain the early onset of the earlier peak in intracellular cryptococci. This indicates that high intracellular growth can also be a protective mechanism to the host earlier in the infection, as there is limited tissue damage from extracellular growth. The early limitation of damage to the host tissue through intracellular growth can also contribute to the pathogenesis as reduction in pro-inflammatory immune signaling, thus allowing for the cryptococcal infection to become fixated in the host [36]. In another study conducted by Garelnabi et al. [38], where blood from healthy donors was analyzed for macrophage interactions with *C. neoformans*, it was found that intracellular proliferation varied between individuals. This finding suggests that the underlying basis for variation lies elsewhere in the genome or occurs at the level of environmental stimuli. As this study analyzed the blood of healthy individuals, it could be a useful benchmark for future research and treatment plans in immunocompromised individuals, since the natural variation in macrophage responses are crucial to understand [38]. Once internalized by macrophages, *C. neoformans* transcriptional profile undergoes significant changes. Following internalization, genes involved in oxidative stress, hexose transporting, mating, and lipid metabolism were found to be upregulated, as well as others. Downregulated genes included those involved in ribosome functions, translation, and rRNA processing, suggesting a decrease in protein production in the yeast following phagocytosis [39]. Studies have also shown that *C. neoformans* affects macrophage gene expression and function. When *C. neoformans* releases extracellular vesicles, it can stimulate macrophages to produce cytokines and nitric oxide, increasing the macrophages’ antimicrobial activity [40]. Once inside the macrophage, *C. neoformans* changes the phagolysosomal permeability, allowing for its survival inside the macrophage [17]. Overall, there is a complex interaction between *C. neoformans* and host macrophages, and this interaction plays a crucial role in the control of the pathogen and disease outcome.

### 2.6. Conclusion

**Figure 1:**
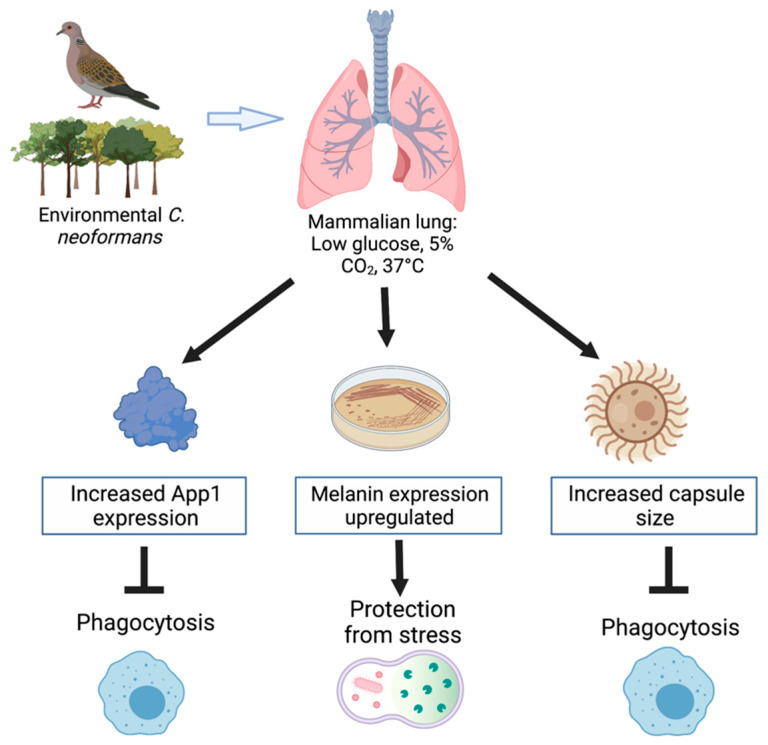
The mammalian lung environment alters the expression of virulence factors resulting in protection from the host’s immune response. Figure created with BioRender.com.

The various virulence mechanisms used by *Cryptococcus neoformans* contribute to its increased resistance to the host’s immune response. It is critical to understand these virulence factors, as the interactions of the pathogen with host cells is important for the control and treatment of the infection. The expression of these virulence factors is affected by conditions of the human host, as summarized in Figure 1. The development of the polysaccharide capsule, formation of melanin, secretion of App1, and host–pathogen interactions allow for the inhibition of phagocytosis and protection from stress, thus allowing for fungal growth. The polysaccharide capsule of *C. neoformans* is able to expand in response to the environmental stimuli it is presented with. Environmental factors, such as CO_2_ concentration, pH levels, temperature, and iron acquisition, can all induce the formation of a capsule. Exposure to CO_2_ in a variety of mammalian sera induced capsule formation [14,18]. Studies have also indicated that capsule induction can greatly increase in environments with a pH greater than 7, indicating that capsule growth occurs in more alkaline mediums [14,15]. Interestingly, the impact temperature has on capsule growth has been discovered to be dependent on the strain of *C. neoformans* present. In contrast, capsule size has been found to be reduced in vps23 and rim101 mutant strains of *C. neoformans* due to iron-related growth defects, resulting in a reduced cell-associated capsule, increased capsule shedding, and increased sensitivity to the alkaline pH [19]. Similarly, melanization also occurs in *C. neoformans* cells as a virulence factor, which is also impacted by many environmental factors such as, temperature, stress (ROS/RNS), and exposure to heavy metals.

The production of App1 has been found to be expressed in various situations, depending on the fungal cell environment and needs [6]. Particularly, App1 expression is upregulated in conditions of the human host lung, which includes 37 °C, 5% CO_2_, and low glucose levels. App1 has been found to be regulated throughout the process of infection; however, in tested fluids it has been found that App1 expression was highest in the bronchoalveolar lavage (BAL) and the lowest in blood serum [6]. The host–pathogen interactions upon infection are crucial to understand, as the yeast has been able to develop virulence mechanisms, even after it has been engulfed by host phagocytes. Additionally, the adherence of phagocytes was shown to be reduced as early as an hour after capsule formation, indicating that adherence allows for the internalization by host phagocytes of *C. neoformans* cells [37]. Host macrophages have also been discovered to be essential for the control of the pathogen, as their depletion has been found to have a negative impact on the restriction of fungal burden [36].

The discussed virulence factors of *C. neoformans* have been shown to increasingly allow for the adaption of the fungus depending on the environmental effects. Growth of the fungus is upregulated secondary to the virulence factors of *C. neoformans*. The understanding of these virulence factors is critical to understand in order to create possible treatments in efforts to control the pathogenesis of the yeast. Although there are many ways the yeast can respond to environmental conditions, it has also been found that the environmental parameters impact the efficacy of antifungals, which will be further discussed in this review.

## 3. Environmental Effects on Antifungal Susceptibility

Cryptococcosis is treated using several common antifungal therapeutics, including AMB, 5-FC, and FLU. Although there are variations based on the location and severity of disease as well as the immune state of the host, common treatment protocols include induction therapy with the combination of AMB and 5-FC, followed by consolidation and maintenance therapy with FLU [2]. Previous theories suggested that AMB killed fungi by creating pores in the membrane, resulting in cellular leakage and cell death [41]. Recently, AMB has been shown to elicit its antifungal properties by extracting ergosterol from the fungal membrane, removing an essential membrane lipid [42]. Resistance to AMB is rare; however, due to toxicity, the use of AMB is limited [41]. Fluconazole exerts its antifungal effect by blocking ergosterol synthesis, specifically by inhibiting the enzyme encoded by ERG11. The lack of ergosterol, combined with the addition of a toxic sterol in the fungal membrane, results in limited cell growth. Fluconazole is therefore considered to be fungistatic against *C. neoformans* [41]. Flucytosine has a different mode of action as it works by mimicking cytosine in the cell, resulting in the inhibition of DNA and RNA synthesis [43]. Common treatment protocols for Cryptococcal meningoencephalitis include two weeks of a combination of AMB and 5-FC, followed by therapy with FLU for often over a year [2]. Antifungal resistance abilities have evolved as fungi have aimed to adapt and survive environmental changes [41]. The frequency of mutations leading to antifungal resistance varies for each type of drug. Some mechanisms that lead to resistance include limiting drug accumulation in the cell, cell stress activation, or changing the drug’s target [41]. Various factors, including environmental conditions and virulence factors, have recently been shown to play a role in the susceptibility profile of *C. neoformans* to antifungal drugs.

### 3.1. The Role of the Environment and Virulence Factors on Antifungal Resistance

Many environmental conditions and virulence factors have been shown to play a role in antifungal resistance in *C. neoformans*. Melanin, a significant virulence factor of *C. neoformans*, has been shown to affect susceptibility to antifungal drugs, with data dating back to 1994. Wang and Casadevall [44] demonstrated that cells grown in L-dopa, which induces melanin production, are more resistant to AMB compared to non-melanized cells. These data were further supported with the findings that *C. neoformans* becomes more resistant to both AMB when melanin is produced [45]. This was demonstrated using killing assays, which showed less killing of melanized cells treated with AMB compared to non-melanized cells. Authors did not find differences in the MIC calculations using standard NCCLS susceptibility protocols; however, this is explained by the inability to induce melanized cells in the specific media conditions needed for this method. In addition, the authors discovered that AMB binds to melanin. The binding of AMB to melanin may lead to the decrease in the immune modulating effects of both AMB and melanin, and therefore an increase in the survival of *C. neoformans*. These findings may explain the decreased efficacy of AMB in immunocompromised individuals [45]. Further proof of these findings was demonstrated by Ikeda et al. [46], who showed that melanized cells have increased survival following exposure to AMB and FLU, when compared to non-melanized cells. In combination to melanin effects, the age of the *C. neoformans* cells also affects the susceptibility to AMB, with older melanized cells having increased resistance compared to younger melanized cells [29].

CO_2_ is an important environmental parameter that can affect *C. neoformans*, as the fungus encounters atmospheric CO_2_ of 0.04% in the environment and transitions to 5% CO_2_ when it enters mammalian tissues [47]. Increased CO_2_ induces capsule formation, inhibits mating, and slows cell growth. The MIC for AMB is not affected by a transition to 5% CO_2_, but the MIC for FLU did decrease 8- to 10-fold in 5% CO_2_ [47]. CO_2_ may affect membrane fluidity as well as inhibition of important synthesis reactions. These may be potential mechanisms for the antifungal property of CO_2_. Another potential mechanism is that CO_2_ affects one of the virulence factors of *C. neoformans* that is known to affect antifungal susceptibilities (i.e., capsule or App1)

Capsule production has been shown to affect susceptibility to antifungal drugs in vitro [48,49]. Zaragoza et al. [50] demonstrated that the acapsular strain Δcap59 has lower MICs of AMB and FLU. The authors also showed that cells with larger capsules have higher survival rates when treated with increasing doses of AMB. Vitale et al. [48] showed a similar relationship between antifungal susceptibility and capsule size. When cells were grown in capsule-inducing conditions, the resulting MIC of FLU, AMB, and 5FC increased, suggesting the increase in capsule results in a decrease in susceptibility to these drugs. These findings are significant because when the organism is in certain body locations, where a capsule is induced, such as the CSF, antifungal drugs may be less effective. An additional link between nutrient supply, the capsule, and antifungal susceptibilities was discussed by Bosch et al. [51]. In this study, low nitrogen availability resulted in increased capsule production, and decreased susceptibility to AMB and FLU. These capsule studies are great examples of the interaction between the environment, virulence factors and antifungal susceptibilities.

App1 is another virulence factor that has recently been demonstrated to affect antifungal susceptibilities to antifungal drugs [52]. A recent study by Ghaffar et al. [52] showed that the presence of App1 decreased the survival of *C. neoformans* when exposed to AMB and FLU, as the Δapp1 strain showed higher survival rates. This study is interesting as the expression of App1 has been shown to be regulated by the environment [6]. Specifically, App1 is upregulated when *C. neoformans* is exposed to glucose deprivation, 5% CO_2_, and 37 °C [6]. The effect of CO_2_ is interesting, as it would be expected that in 5% CO_2_, both App1 and capsule production would be upregulated [6,47]. The upregulation of both virulence factors is intriguing as they have opposite effects on antifungal susceptibility. Since an increase in CO_2_ does not affect AMB susceptibility, it may be that the change in either virulence factor is not significant enough to exert its effect on susceptibility. Potentially for App1 to increase susceptibility, more environmental conditions may need to be altered. Glucose starvation seems to be the strongest contributor to the environmental-induced upregulation of App1 [6]; therefore, it may be interesting to see if a glucose starvation environment alone would result in a change in antifungal susceptibility. This would be clinically significant, since the mammalian lung environment is a low-glucose environment [6]. Since the lung plays a critical role in the containment of Cryptococcal disease, the susceptibility of *C. neoformans* to antifungal drugs in the lung environment is important for disease outcome. Recently, Carlson et al. [53] studied the effects of environmental stress on the antifungal sensitivities of *C. neoformans*. The authors found that nutrient deprivation leads to a decrease in the sensitivity to FLU, and an increase to AMB. Also, increased temperature increased the efficacy of both FLU and AMB. There appears to be a complex web of interactions between environmental conditions, virulence factors, and antifungal susceptibilities. It is important to continue identifying these relationships to develop the most effective treatment protocols. Given the changes in the environment the yeast encounters when it transitions from the environment to the mammalian host, the resulting genetic and phenotypic changes may have considerable effects on clinical outcomes.

### 3.2. Mechanisms Leading to Antifungal Resistance

Despite the advances in preventative treatments against invasive fungal infections, there has been a significant increase in the morbidity and mortality rates in immunocompromised patients. Antifungal resistance in high-risk patients has become more concerning over time, as pathogens such as *C. neoformans* have developed mechanisms leading to their resistance to antifungal treatments. Common mechanisms of resistance include efflux pumps and changes in the expression of the azole target gene ERG11 [54,55]. The duplication of chromosome 1 of *C. neoformans* is linked to significant antifungal susceptibility differences. This finding is not surprising because ERG1 and AFR1 (an efflux pump) are both located on this chromosome. ERG11 is the gene that produces the enzyme target of azole antifungals. When ERG11 is overexpressed, more ERG11 is produced and therefore more FLU would be required to affect the enzymes produced in the cell [56]. Overexpression of AFR1 leads to increased resistance, while a deletion of AFR1 leads to increased susceptibility [54]. AFR1 is an ABC transporter that pumps FLU out of the cell; therefore, an increase in Afr1 would lead to more FLU being pumped out of the cell, and therefore less effect of the drug [54,57,58]. In addition to AFR1, other efflux pumps have been investigated to determine their potential roles in antifungal resistance. PDR1 and MDR1 have been studied, and Yang et al. [56] found PDR1 to play a role in FLU efflux, therefore affecting resistance. MDR1 has not been found to affect FLU susceptibility [54,56].

Although AMB is widely used to treat Cryptococcosis, resistance is not common. Mutations in the enzymes involved in ergosterol synthesis were identified as playing a role in AMB resistance, and resulted in reduced ergosterol content [59]. Fortunately, this is not a common clinical finding, and *C. neoformans* susceptibility is consistent.

### 3.3. Effect of Antifungal Drugs on Virulence Factor Expression

We have discussed the effect virulence factors have on the antifungal susceptibility of *C. neoformans*. However, there appears to be a two-sided relationship between these two variables. Studies have begun to elucidate the effects antifungal exposure has on the gene expression of *C. neoformans* and its virulence factors. Florio et al. [60] performed microarray analysis to identify gene expression changes following exposure to FLU. The results of this study showed 476 genes were differentially expressed in response to FLU, with 231 upregulated and 245 downregulated. Many studies have since been done to examine specific effects on gene expression following antifungal exposure. Interestingly, AFR1 was found to be upregulated in response to FLU [54]. Therefore, in response to FLU exposure, *C. neoformans* will upregulate AFR1, which will lead to the ability to pump the drug out of the cell and increase resistance [54].

Virulence factors have also been shown to be affected by antifungal exposure. Recently, the expression of App1 was shown to be upregulated by FLU and downregulated by AMB [52]. As discussed previously, App1 increases the susceptibility of *C. neoformans* to both FLU and AMB [52]. In addition, an earlier discussion included the finding that nutrient deprivation and increased temperature changed antifungal susceptibilities, and these factors also affect App1 expression. Temperature and nutrient deprivation increase App1 expression, and App1 expression increases the efficacy of FLU; however, Carlson et al. [53] found that nutrient deprivation decreases the efficacy of FLU. These findings are contradictory and deserve more attention. There are various factors at play, including the degree of nutrient deprivation, relative change in App1 expression, and antifungal dose and time of exposure. The findings of AMB are different, as nutrient deprivation was found to cause an increase in sensitivity to AMB. App1 could be the factor affected by nutrient deprivation that then affects AMB sensitivity. This complex interaction between the antifungal drugs, the environment, and App1 deserves further studies.

The polysaccharide capsule of *C. neoformans* has also been found to be affected by both AMB and FLU. The capsular appearance was altered and more glucuronoxylomannan was released from the cells following exposure to the antifungal drugs [61]. A study by Martinez et al. [62] showed that FLU and AMB did not affect melanin production. There appears to be an effect of antifungal drugs on some of *C. neoformans* virulence factors, and these virulence factors also affect the susceptibility of *C. neoformans* to the antifungal drugs. These intricate relationships deserve further studies in an effort to determine the most effective treatment protocols based on a patient’s immune status, location of infection, and disease severity.

### 3.4. Conclusions

**Figure 2:**
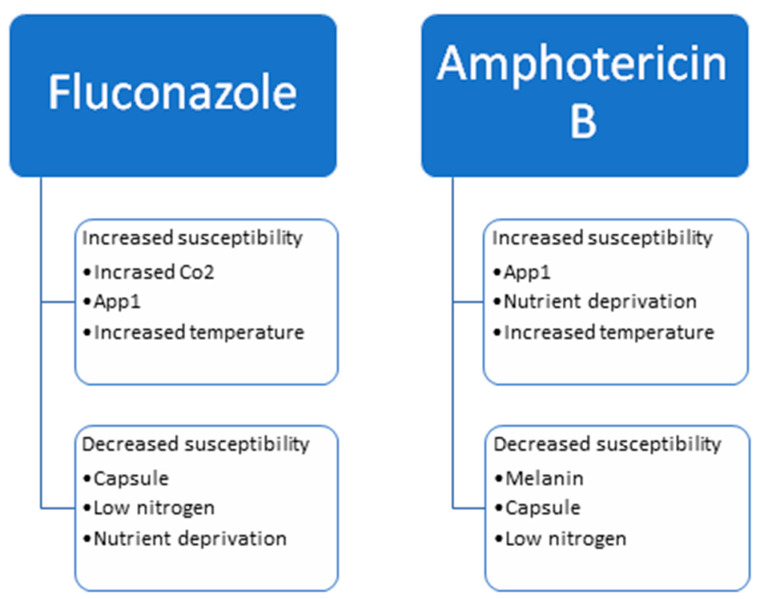
Factors that influence the susceptibility of *Cryptococcus neoformans* to fluconazole and amphotericin B.

Current treatment for Cryptococcosis relies on AMB and 5FC, followed by maintenance therapy with FLU. Variations in the protocol are dependent on the immune status of the patient, the location of the disease, and the severity of the disease. Understanding the role the environment and virulence factors have on antifungal susceptibility helps our understanding of the best treatment protocols. Melanin, capsule and nitrogen deprivation lead to greater antifungal resistance, whereas CO_2_ and App1 lead to greater antifungal susceptibility. Figure 2 depicts the factors leading to the increased resistance or susceptibility to FLU and AMB. Studies examining the mechanisms for antifungal resistance point to a change in ERG11, the gene for the drug’s target, as well as efflux pumps including AFR1 and PDR1. Many studies have examined the effects virulence factors have on antifungal susceptibility, but it is also important to examine the effects antifungal drugs have on virulence factors. FLU and AMB have been shown to affect App1 and capsule production. Transcriptional analyses have shown that FLU leads to transcriptional changes in hundreds of genes.

These relationships are important and may lead to individualized treatment plans. For example, App1 is a virulence factor that leads to decreased phagocytosis and therefore faster disease progression. However, in an immunocompromised host, a decrease in phagocytosis actually slows disease progression because, once phagocytosed in these hosts, the yeast is not killed and therefore grows undetected inside the phagocyte. For this reason, decreasing phagocytosis is actually beneficial in an immunocompromised host. App1 also increases the efficacy of FLU and AMB. This is only one example of the importance of understanding how antifungal drugs and virulence factors are related. The antifungal drug choices may need to be individualized per patient based on their immune status and disease characteristics.

## 4. Conclusions and Significance

The pathogenic fungus, *Cryptococcus neoformans*, has been recognized as a common initiator of fungal meningoencephalitis, which has been identified as a public health burden for many years. The yeast is able to impact immunocompromised individuals, especially those with HIV/AIDS. The various virulence factors discussed in this review, which include capsule formation, melanin secretion, and antiphagocytic protein 1, all aid in the survival of this fungus in host cells. These virulence factors allow *C. neoformans* to evade the host’s immune response and further multiply within the host. The continued resistance of the yeast has allowed for its susceptibility to be decreased towards antifungals such as fluconazole, flucytosine, and amphotericin B. The pathogenicity of *C. neoformans* has been greatly increased secondary to the virulence factors associated with it; however, there are many environmental factors impacting them that enable *C. neoformans* to successfully adjust to unpredictable environments, which we have discussed in this review. As *C. neoformans* transitions from its environmental reservoir to the human host, the expression of virulence factors changes, which can affect the outcome of disease.

The polysaccharide capsule of *C. neoformans* can be induced under many different environmental conditions, which include exposure to CO_2_, alkaline pH levels, temperature (37 °C), and exacerbation of iron. Melanin is another virulence factor of *C. neoformans* that is able to assist in the survival of the fungus, as it protects against the oxidants produced by immune cells and decreases the permeability of the cell wall. Melanin accumulates in the cell wall of *C. neoformans* to protect against many types of stressors. Melanin production can be affected by temperature, exposure to reactive nitrogen and oxygen species (RNS and ROS), exposure to heavy metals, and in the presence of diphenolic compounds (L-dopa).

Antiphagocytic protein 1 (App1) is a protein secreted extracellularly by *C. neoformans*, which inhibits attachment and ingestion of the yeast through the inhibition of phagocytosis. Interestingly, it was discovered that the effect of App1 on Cryptococcosis is dependent on the immune state of the host. App1 has been found to be upregulated in conditions of the human host lung, which include 37 °C, 5% CO_2_, and low glucose levels. The expression of App1 was found to be greatest in the bronchoalveolar lavage (BAL), where the protein is most influential, and lowest in the blood serum, where the protein would have a decreased effect. Host–pathogen interactions are found to also have a critical role in the infectious process of the fungus, aiding in its virulence. Host macrophages have been found to be essential for the control of the pathogenesis of the fungal infection, as the reduction in macrophages has been indicated to have a damaging impact on the restriction of fungal burden.

Treatment against the fungal pathogen includes dependence on the antifungal drugs amphotericin B and 5-fluorocytosine, followed by maintenance therapy with fluconazole. However, as *C. neoformans* can readily adjust to environmental changes through its virulence factors, there has been found to be decreased susceptibility to these antifungal treatments. FLU and AMB have been shown to have an impact on App1 and capsule production. It is important to realize the relationships between these treatment methods and their impact on virulence factors, in order to better understand how treatment plans can be individualized depending on each patient’s immune status and the characteristic of the disease.

## Data Availability

Not applicable.
